# Whole Genome Sequencing of Lumpy Skin Disease Virus from 2021–2023 in Eastern Eurasia Reveals No More Recombination Signals in the Circulating Pool of Strains

**DOI:** 10.3390/v17040468

**Published:** 2025-03-25

**Authors:** Alexander Sprygin, Alena Krotova, Ma Jun, Olga Byadovskaya, Vladimir Kirpichenko, Jinchao Chen, Tserenchimed Sainnokhoi, Ilya Chvala

**Affiliations:** 1 Federal Center for Animal Health, Vladimir 600901, Russia; krotova@arriah.ru (A.K.); byadovskaya@arriah.ru (O.B.); chvala@arriah.ru (I.C.); 2 Kazakh Scientific Research, Veterinary Institute, Almaty 050016, Kazakhstan; majun@fosu.edu.cn (M.J.); vlad_92reik@mail.ru (V.K.); 3 Guandong Provincial Key Laboratory of Animal Molecular Design and Precise Breeding, School of Animal Science and Technology, Foshan University, Foshan 528225, China; 4 State Central Veterinary Laboratory, Ulaanbaatar 17024, Mongolia

**Keywords:** capripoxvirus, lumpy skin disease virus, phylogeny, evolution, molecular epidemiology

## Abstract

Having spanned thousands of kilometers from Africa through Europe, the Middle East, Central Asia through to the south eastern part of Eurasia in the recent decade, lumpy skin disease virus has now become entrenched in China, Thailand, Vietnam, and South Korea. In light of discovered findings on recombination, cluster 2.5 lineage strains are now dominant and continue to spread throughout Southeast Asia. To gain a better picture of the phylogenetic landscape in the field, whole genome sequencing of 11 LSDV isolates from Russia and Mongolia collected from 2021 to 2023 has been attempted to see the dynamics of recombination signals, as was shown for LSDV circulating in 2017–2019 in Russia and Kazakhstan. Deep sequencing performed direct from skin nodules along with data retrieved from Genbank provides the most recent update on molecular epidemiology of LSDV and demonstrates that no more mosaic variant of LSDV has been observed, and cluster 2.5 lineage is now the dominant lineage currently on the rise in the region with its own patterns of monophyletic evolution. These discoveries may help future investigations aimed at epidemiological surveillance and virus tracking in the context of currently identified lineages worldwide.

## 1. Introduction

Lumpy skin disease virus remains a continuing threat to the cattle industry worldwide [1]. The annual losses caused by LSD can reach USD 91.33 million, which places a heavy burden on farmers. For this reason, LSD is bound to be reported to the World Organization of Animal Health (WOAH), followed by trade restrictions and quarantine [2].

The aetiological agent is a poxvirus from the genus Capripoxvirus that includes sheep pox virus and goat pox virus [3,4]. The genome length is about 150 kbs and contains 156 ORFs [5].

Typical symptoms of LSD include fever, nasal and ocular discharge, lymph node enlargement, and nodules—distinctive raised cutaneous bumps from 0.5 to 5 cm in diameter that grow over 3 to 4 days from first roseolas to skin lumps, followed by necrosis, sequestration, and sloughing off in about 14–21 days post appearance [6,7,8].

LSDV has host tropism toward not only cattle and water buffaloes but also wildlife species like springbok, Oryx, and giraffe, and experimentally from impala [9,10]. LSD was recently documented in camels and free-ranging Indian gazelles (*Gazella bennettii*) once the disease occurred in India in 2022 [11,12] and in yaks in China [13], and in gaurs (*Bos gaurus*), Mainland serow (*Capricornis sumtraensis*), and banteng (*Bos javanicus*) in Thailand [14,15].

LSDV transmission has enjoyed much focus recently due to the dearth of knowledge in the face of vast range expansion since 2015 [16]. Although sheep pox virus and goat pox virus can spread via contact and insect bites, the primary mode of LSDV spread was through the bite of hematophagous arthropods following rainy seasons or within hot months, with the contact mode deemed ineffective [17,18]. With the emergence of naturally occurring recombinant LSDV strains, the contact mode proved true, with outbreaks occurring during cold and snowy months [19,20,21].

From the prospect of global epidemiology, following the incursions of LSD into countries of the Northern Hemisphere like Russia, Serbia, and Bulgaria in 2015–2016, the genetic studies into LSDV diversity enjoyed focused attention and helped with recognizing two major clusters 1.1, including attenuated vaccine strains from commercial vaccines and a virulent strain that circulated in South Africa before 1991, and 1.2 that comprises strains from Israel, Warmbaths (RSA after 1991), Dagestan/2015, Neethling 2490, and KSGP strain, etc. [22,23,24]. Recently, novel clusters representing naturally occurring recombinant vaccine-like strains of LSDV have appeared. In 2017–2021, molecular epidemiological studies reported in the chronological order Saratov/2017 cluster 2.1, Udmurtiya 2019 cluster 2.2, Kostanay/2018 cluster 2.3, Tyumen/2019 cluster 2.4, in China, Vietnam, Mongolia, and Thailand cluster 2.5 and Kurgan /2019 cluster 2.6 [25,26,27]. However, later on, it was presumably discovered that recombinant vaccine like strains were no longer detected in outbreaks past 2019 and the circulating pool seemed to be dominated by only 2.5 cluster lineage in the Southeast Asian countries in contrast to India, where KSGP-like strains are spreading, which is not considered a monophyletic continuation of the epidemic in Southeast Asia [28].

In parallel to the expansion of naturally occurring recombinant LSDV in Southeast Asia, in 2019 LSD broke out in India and Bangladesh, with LSDV strains being reported in the Indian subcontinent as of 2019 that are related to the Kenyan-type LSDV vaccine strains, which indicates an independent spillover of virus into Indian cattle [29,30]. Taking into account that cattle slaughter is opposed by various Indian religions and LSDV-infected cows shed virus for a long time until recovery or death. This is a great hurdle and challenge to curb the disease in the country.

The existence of two lineages in China and India and consequent multiple LSD cases spreading across countries of the region, including Bhutan, Myanmar, Nepal, Hong Kong, Vietnam, Taiwan, and Sri Lanka raise key questions pertaining to the ways the particular lineages spread [30]. Considering that LSDV spreads like other capripoxviruses, i.e., via contact and arthropods, control strategies need revision on a regular basis to ascertain the soundness of eradication measures [31].

Since molecular epidemiological studies provide valuable epidemiological information and tools to track the clonal radiation of a virus in a given territory or detect novel unrelated incursions or spillovers of different lineages, as is the case with LSD in 2017–2019 in Russia [16,32], whole genome sequencing has remained the method of choice to answer important epidemiological questions [33].

The goal of this study is to gain insights into the circulating LSDV isolates from the Far East of Russia in 2021–2022 and to see if the cluster 2.5 has taken over as the dominant lineage without new recombinant events occurring.

## 2. Materials and Methods

### 2.1. Strains

Ten virulent LSDV samples in the form of skin nodules were obtained from active outbreaks between 2020 and 2022 for complete genome sequencing at FGBI ARRIAH (Vladimir, Russia). The samples analyzed during this study were such that they were the only samples available in the form of nodules amenable for downstream whole genome sequencing. Sequencing was performed with DNA extracted directly from clinical samples without prior propagation on cell culture. The brief sample data are summarized in Table 1.

**Figure 1:**
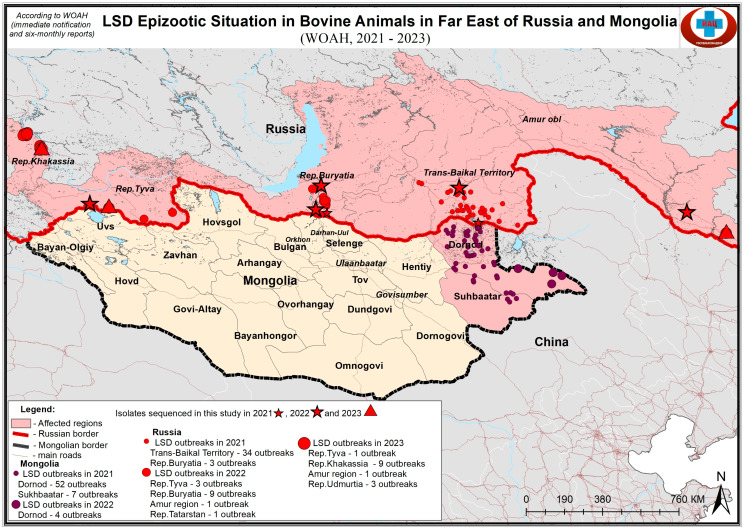
Map showing the sampling sites of LSDV samples in Eastern Eurasia. Due to the limited map space, the sample udm23 (Table 1) is not shown.

The location of outbreaks is shown in Figure 1.

### 2.2. Sequencing

Total genomic nucleic acid (DNA) was extracted using Trizol (Invitrogen) as previously described. Prior to DNA extraction, tissue samples (nodules) were crushed with a sterile grinder, and a 10% (*v*/*w*) suspension with phosphate-buffered saline (Invitrogen, Carlsbad, CA, USA) was prepared.

Then, 200 ng of purified DNA from nodules was fragmented into 100–700 bp fragments, with a peak distribution between 250 and 300 bp, using a Covaris ME220 focused-ultrasonicator (Covaris, Woburn, MA, USA) following the manufacturer’s instructions. A total of 300 bp fragments was purified using magnetic beads (SPRI), and 25 ng of DNA was processed according to the MGIEasy Universal DNA Library Prep Set (MGI Tech, Shenzhen, China) protocol. The latter entails blunt-end polishing of DNA fragments and ligation to 10 bp single-end indexes adapters (MGI Tech, Shenzhen, China).

The DNBSEQ-G50 platform (MGI Tech, Shenzhen, China) and pair-end 150 sequencing protocol were used to generate datasets. Each sample yielded datasets with 10–100 million paired reads.

### 2.3. Bioinformatics

The FASTQC quality control tool was utilized to ascertain high throughput sequence data quality, followed by filtering and cleaning with PRINSEQ by removing 3 bases from the 5’ end and 5 bases from the 3’ end, with a minimal length of 25 [34]. Quality reads were mapped against the genome of the China/GD01/2020 strain sequence (Cluster 2.5) (GenBank accession number: MW355944) using bowtie2 under default parameters and the--*no*-*unal* optional argument on [35]. The consensus sequence was called with bases that make up at least 90% of the depth at a position and the minimum depth to call consensus was 400 to ascertain the high-quality analysis.

### 2.4. Phylogenetic Analysis

To investigate the phylogenetic relatedness, a total of 97 whole-genome sequences representing virulent isolates and vaccine strains were aligned with the 11 newly generated consensus sequences reported herein collected during the 2021–2023 period of LSDV epidemic.

Whole genome sequences were aligned using MAFFT [36] with auto settings and automatically trimmed with trimAl 2.rev0 build 2019-08-05 [37]. The best-fitting DNA substitution models were selected using ModelTest-NG [38]. The aligned sequences and selected DNA substitution models were used for ML analyses, and ML trees were reconstructed using Randomized Accelerated Maximum Likelihood (RAxML) algorithm to infer phylogenetics with 1000 bootstrap replicates [39]. The ML distances were visualized with Chiplot v 2.6.1 [40]. Single nucleotide polymorphism (SNP) differences were extracted using SNP-sites package [41].

## 3. Results

Forward and reverse reads for each isolate analyzed accounted for 100 to 150 million reads, of which 1–3% proved to be on-target reads. The average coverage of the mapped on-target reads ranged between 610 and 844 across the analyzed samples, resulting in the generation of a single consensus sequence per sample. The obtained consensus assemblies were deposited in GenBank under the accession numbers PQ727625-PQ727635.

**Figure 2:**
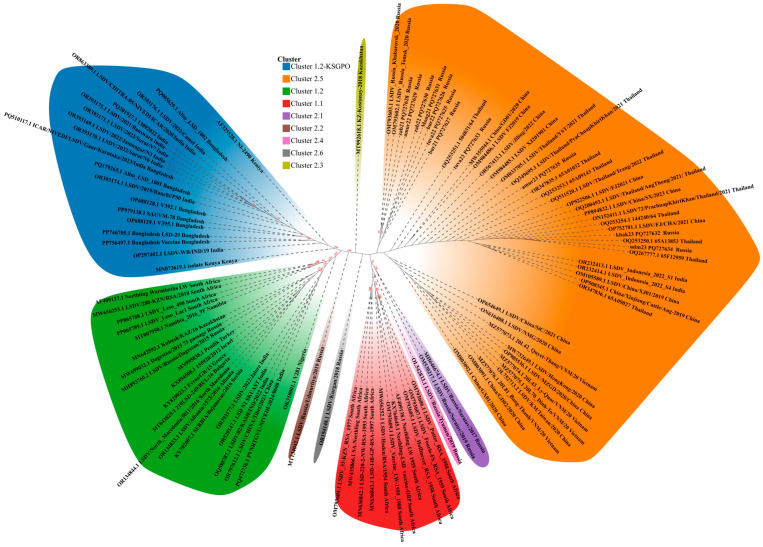
WGS-based phylogenetic ML tree showing relationship between the isolates of this study and those available in Genbank.

The phylogenetic inference placed the 96 available isolates into clusters ranging from 1.1 and 1.1 up to 2.6—a total of eight clusters, highlighted in different colors for clarity (Figure 2). Cluster 1.1 (red) includes archived LSDV field isolates, for example OM793605.1 LSDV_Hoffmeyer_RSA_1958 (in circulation in South Africa before 1990) and commercial live vaccine strains like KX764645 Neethling-LSD vaccine-OBP. Cluster 1.2 (green) is split in two: cluster 1.2 with classical field isolates found in Africa, Middle East, Turkey, Serbia, Southern Europe, Russia (prior to 2017), such as AF409137.1 Neethling Warmbaths LW (South Africa), PP065788.1 LSDV_Leso_490 (Lesotho, Africa), MN995838.1 Pendik (Turkey), KY702007.1 SERBIA/Bujanovac/2016 (Serbia), OR134833.1 LSDV/Albania/1573/2016 (Albania), R134844.1LSDV/North_Macedonia/5011/2016 (Northern Macedonia), MH893760.2LSDV/Russia/Dagestan/2015 (Russia), OR393177.1 LSDV/2022/Jalore (India), OR520147.1LSD N1 SKUAST (India), OR797612.1 LSDV/CHINA/Tibet/2023 (China) and Cluster 1.2 Kenya-like (blue) Kenya PQ179265.1Alim_LSD_1001 (Bangladesh), OR393176.1 LSDV/2022/Camel (India), OR863389.1LSDV/CHITRA-05/NIVEDI/ICAR/2020/India (India), MN072619.1 isolate Kenya (Kenya), etc.

The representatives of Cluster 1.2 (green) exhibit from 1 to 65 nucleotide differences across the group, while within Cluster 1.2 (green) there exists a subgroup including strains from India and China Tibet with OR393177.1 (India), OR520147.1 (India), OQ588787.1 (India), OR797612.1 (China Tibet) being identical, whereas PQ472736.1 has two nucleotide differences from the said subgroup.

The representatives of Cluster 1.2 (blue) demonstrate from 1 to 7 nucleotide differences excluding AF3225528.1 NI-2490 Kenya, which has from 5 to 11.

Cluster 2.1 to Cluster 2.6 except for cluster 2.5 are formed by single representatives of naturally occurring recombinant LSDV strains in Russia and Kazakhstan from 2017 to 2020 MH646674.1 LSDV/RUSSIA/Saratov/2017 (cluster 2.1, Russia), MT134042.1 LSDV/RUSSIA/Udmurtiya/2019 (cluster 2.2, Russia), MT992618.1 KZ-Kostanay-2018 (cluster 2.3, Kazakhstan), OL542833.1 LSDV/RUSSIA/Tyumen/2019 (cluster 2.4, Russia), OM793602.1 LSDV_RUSSIA_Tomsk_2020 (cluster 2.5, Russia), OR194148.1LSDV/Kurgan/2018 (cluster 2.6, Russia) (Figure 2).

All 11 sequences analyzed during this study isolates fell into cluster 2.5 together with LSDV_Indonesia_2022_S1 (Indonesia), LSDV/KM/Taiwan/2020 (China), LSDV/Thailand/PraChuapKhiriKhan/2021 (Thailand), 20L81_Bang-Thanh/VNM/20 (Vietnam), LSDV_Russia_Khabarovsk_2020 (Russia), LSDV/Jiling/2022 (Mainland China), etc. (Figure 2).

The Russian isolates formed two sublineages within cluster 2.5: one including OM793603 Khabaraovsk/2020, OM793602 Tomsk/2020, zab2021 and Amur2022 with 99% bootstrap support, the other sublineage containing zab22 PQ727630, Mong2021 PQ727631, bur22 PQ727626, tuva22 PQ727625, bur21 PQ727627 with 97% bootstrap support (Figure 2).

The isolates tuva23 PQ727633, amur23 PQ727635, udm23 PQ727634, and khak23 PQ727632 grouped together with the majority of isolates from cluster 2.5.

The genome wide genetic identity within cluster 2.5 varied from 0 to 97 SNPs, whereas within the sequenced isolates from 0 to 13 SNPs. The most divergent representative of cluster 2.5 is MW732649 HongKong/2020 China with 72–97 SNPs as compared to others within the cluster (Supplementary File S1).

The extracted SNPs are given in a variant call format (VCF) in Supplementary File S2.

## 4. Discussion

Following the waves of LSD outbreaks in the northern hemisphere, the interesting and important insight was for the first time uncovered—the potential for recombination [26]. Although it was a theoretical speculation in the past [41], the occurrence of recombinant vaccine-like strains was precipitated by a contaminated vaccine [42,43]. Since the vaccination lasted for a few years, almost every outbreak in Russia in 2017–2019 was attributable to a unique mosaic virus belonging to a novel previously unestablished lineage that gave rise to a plethora of new clusters behind 2.0 [25], whereas outbreaks in Russia that occurred within the 2015–2016 period as well as in other countries maintained clonality and aligned solely with cluster 1.2 [44]. Notably, genome conservatism is typical of capripoxviruses [4,45].

Currently, cluster 2.5 strains are on the rise in countries in Southeast Asia like China, Thailand, Vietnam, and South Korea [22,46,47,48]. The first representative of cluster 2.5 occurred in China in 2019 25 km off the border of Kazakhstan, while in Russia that was the only country where recombinant strains were in circulation, had never reported that lineage before [48]. Only months later, cluster 2.5 strains were documented in Russian regions close to Kazakhstan and China [26].

The circulation of cluster 2.5 recombinants may necessitate the validation of the use of vaccines currently in use in endemic regions [49,50]. Because recombinant LSDV demonstrates altered and even more aggressive features, these issues need further consideration, but the first efforts in this direction have already been taken, showing the efficacy of a Neethling vaccine against cluster 2.5 strains [51,52].

In this study, we performed whole genome sequencing of a LSDV strain recovered from outbreaks in 2020–2022. We successfully generated viral reads from nodules without resorting to culture enrichment by increasing read count per sample using the DNBSEQ-G50 platform, as already successfully demonstrated for other capripoxvirus-containing samples [53].

Although in 2019 cluster 2.5 was already dominating in the field, having displaced the previously reported lineages, it was epidemiologically important to investigate where the recombinant strains are still occurring and to which clusters they belong [22,54].

The obtained findings clearly demonstrate that the sequenced strains fall into cluster 2.5, lending support that we earlier expressed in our previous studies that cluster 2.5 established in the region, and no more recombinant strains are to be expected [25,33]. Interestingly, the cessation of recombinant strain occurrence in 2019 onwards timed with the discontinued mass administration of Lumpivax (Kevapi, Kenya) in Kazakhstan cattle. That vaccine was proved contaminated with a mixture of recombinant capripoxviruses [43], which generated multiple recombinants spilling over into Russian cattle in 2017–2020 [26]. The strains Kurgan/2018 and Kostonay/2018 are exemplary in this case (Figure 2). Interestingly, according to the manufacturer’s information, the safety profile of said vaccine was evaluated in guinea pigs rather than the natural host-cattle.

Since a plethora of strains were in circulation like Saratov/2017, Udmurtiya/2019, Kurgan 2018 etc., only cluster 2.5 strains outcompeted other lineages, and it remains the only dominant lineage in Southeast Asia (Figure 2). There is no doubt, that the ability of the cluster 2.5 lineage to survive and entrench is due to yet unknown viral fitness features favored by climatic factors and naïve hosts in Asia, including wild fauna.

The analyzed sequences shared over 99.99% identity with cluster 2.5, although the overall identity within cluster 2.5 varies from 99.6% to 100% (Supplementary File S1). Although no recombination is evident from the reported data, circulation-driven evolution (Figure 2) was the case for LSDV in Saratov region that had survived two climatic winters in northern latitudes and maintained replication [55]. Notably, there are a few sublineages inside cluster 2.5 (Figure 2).

Cluster 2.5 strains are taking over more regions in Southeast Asia, including South Korea and Japan (https://wahis.woah.org), whereas in India and Bangladesh the KSGP-like virus lineages is distributed, which creates two recognized pools of LSDV circulation with a risk for co-infection at the interface of the said countries; for example, at the shared border of China and India [56,57]. Previous studies lend support to the seasonality pattern of LSDV transmission and spread in the field, where hot months and precipitation favor vector-borne transmission, when populations of arthropods are abundant [26,58,59]. Following the observation of contact transmission in recombinant LSDV strains like Saratov/2017 (cluster 2.1) or Udmurtiya (cluster 2.2) [20,21], the epidemiological situation in Asia with regard to the risks of LSDV spread is alarming in the context of the climate favoring vector abundance and the recombinant virus lineage (cluster 2.5) in circulation capable of contact mode transmission. These environmental factors allow the virus to be efficiently maintained within the South Asian milieu.

Of particular note, Genbank lists entries of whole genome sequences from India (OR393177, Jalore India for example) and China (OR797612 LSDV/CHINA/Tibet/2023) that do not belong to cluster 1.2-Keniya and Cluster 2.5, correspondingly as expected, according to published data [30,56,60], but belong to a distinct sublineage within cluster 1.2 with 100% identity (Figure 2). The geographic range of cluster 1.2 (for example Warmbaths, Dagestam/2015, etc) tends to span across Africa, Europe, Russia from 2015 to 2016, and the Middle East, and has never been reported in China or India so far [11,30]. The appearance of such lineages in those regions cannot be explained by monophyletic evolution and points to human-assisted introduction whether through illegal testing of novel vaccine preparations or spillovers from infected cattle from endemic regions. In this regard, more studies are needed to shed light on these challenges.

The circulation of different lineages amid the established lineage presence raises logical questions regarding the possible ways of introduction and necessitates further study in light of the fact that a particular sublineage did not occur in the region before 2022 [32,33]. Having said that, potential future surveillance approaches would be required to assist in the surveillance and control measures. Considering the availability of molecular tools, this situation should be monitored through the use of the recently published PCR assays capable of differentiating among the current circulating clusters 2.5, 1.2, and 1.1. in tandem with full genome sequencing to shed light on the global LSD epidemiology [61,62]. In regard to the control of the situation with cluster 2.5 dominance in the region (China, Vietnam, Thailand, etc.), homologous Neethling vaccines may be used as they have already shown efficacy for this particular lineage in a controlled environment [52,63]. Heterologous vaccines are also in use against recombinant LSDV [64], so farmers have an informed choice over which vaccination option to pursue. As for India, vaccine studies are warranted to test the available vaccines and their efficacy against cluster 1.2 (Kenya-like).

The findings reiterate the importance of ongoing surveillance for LSDV distribution across countries in Southeast Asia with a focus on lineage determination, which will enable the tracking of virus evolution and the identification of transmission patterns in the environmental conditions of the Asian climate, as well as guide informed vaccine decisions.

## Figures and Tables

**Table 1:** Summary of the sequenced LSDV samples.

Sample Name	Location	Year of Origin	Sample Type
tuva22	Tuva republic, Russia	2022	Skin lesion
bur22	Buryatiya republic, Russia	2022	Skin lesion
bur21	Buryatiya republic, Russia	2021	Skin lesion
zab21	Transbaykal territory, Russia	2021	Skin lesion
amur22	Amur oblast, Russia	2022	Skin lesion
zab22	Buryatiya republic, Russia	2022	Skin lesion
khak23	Khakassia republic, Russia	2023	Skin lesion
tuva23	Tuva republic, Russia	2023	Skin lesion
udm23	Udmurtiya republic, Russia	2023	Skin lesion
amur23	Amur oblast, Russia	2023	Skin lesion
Mong21	Mongolia	2021	Skin lesion

## Data Availability

The datasets presented in this study were submitted to the Genbank database (PQ727625-PQ727635).

## Supplementary Materials

The following supporting information can be downloaded at: https://www.mdpi.com/article/10.3390/v17040468/s1. File S1: Cluster 2.5; File S2: The extracted SNPs.
